# Regulation of arachidonic acid in esophageal adenocarcinoma cells and tumor-infiltrating lymphocytes

**DOI:** 10.3892/ol.2013.1267

**Published:** 2013-03-20

**Authors:** WEI SONG, RUI JIANG, CHUNMING ZHAO

**Affiliations:** 1 Departments of Oncology, Provincial Hospital Affiliated to Shandong University, Jinan, Shandong 250021, P.R. China; 2 Opthalmology, Provincial Hospital Affiliated to Shandong University, Jinan, Shandong 250021, P.R. China

**Keywords:** arachidonic acid, esophageal adenocarcinoma cell lines, tumor-infiltration lymphocytes, regulatory T cell

## Abstract

The generation and development of esophageal adenocarcinoma (EAC) are correlated with neuroimmunological factors. The aim of this study was to observe the effectiveness of the neurotransmitter arachidonic acid (AA) on two EAC cell lines, OE19 and SK-GT-4, as well as three isolated tumor-infiltrating lymphocytes (TIL1, 2 and 3). C-X-C chemokine receptor type 4 (CXCR-4) and tumor necrosis factor receptor 1 (TNFR1) expression, cell migration, necrosis, cytokine secretion and cytotoxicity of TILs were investigated. AA dose-dependently increased the migration of all cells. However, AA did not increase the percentage of cell death of the three TILs in the presence of a necrosis-inducing agent. AA dose-dependently increased the cytotoxicity of the three γδT cell-enriched TILs compared with the OE19 and SK-GT-4 cell lines. AA also dose-dependently increased the secretion of interferon-γ (IFN-γ) and TNF-β in TIL1 and 2. However, the cytokine secretion and cytotoxicity activity of TIL3 and γδT cell-enriched TIL3 were the lowest. Furthermore, the percentage of CD4^+^forkhead box p3 (Foxp3)^+^ regulatory T cells in TIL3 was the highest. The effect of AA on tumor cells and TILs is different. The degree of malignancy of the tumor and the ratio of regulatory T cells may be the main factors determining the function of AA.

## Introduction

Neurotransmitters are not only chemical messengers, but also regulators for the immune system and tumor cells. Several studies have demonstrated the involvement of neurotransmitters in tumor cell progression and metastasis development and also in the migration of lymphocytes (1–3). The chemokine system is essential in tumor cell metastasis and leukocyte trafficking (4). The chemokine stromal cell-derived factor-1 (SDF-1) and its receptor C-X-C chemokine receptor type 4 (CXCR4) are considered the first identified and most notable substances involved in tumor cell migration.

Neurotransmitters have numerous effects on the immune system and tumor cells. They modulate proliferation, apoptosis, angiogenesis and metastasis of cancer by releasing growth factors, angiogenesis and metastasis factors, arachidonic acid (AA), pro-inflammatory cytokines and local neurotransmitters from cancer cells and their microenvironment (5).

AA and other fatty acids regulate neuronal excitability directly, via mechanisms that do not involve metabolism or intervention of other secondary messenger pathways. Three pathways of AA metabolism have been discovered in the majority of animal tissues, including via lipoxygenases, cyclooxygenases and cytochrome P450, which are associated with inflammation. During inflammation, activation of the cytosolic phospholipase A2 (cPLA2) releases AA from membrane phospholipids (6). AA stimulates lymphocyte superoxide production, degranulation and the expression of complement receptors type 3 (CR3) (7,8). In this manner, AA perpetuates the inflammatory reaction.

One study identified that there is an association between cancer and chronic inflammation in adults (9). Such inflammatory processes are often associated with hypermethylation of promoter regions in tumor-suppressor and/or pro-apoptotic genes (10). Furthermore, following this acquisition of genetic limitations in apoptotic pathways, the resultant increase in necrotic cell death leads to the release of cellular contents, which in turn promotes cell growth, cancer progression and tumor-infiltrating leukocyte recruitment (11). The cell necrosis pathway is determined by one key mitochondrial phosphatase, PGAM5, which is at the convergence point of multiple necrotic death pathways (12).

Tumor necrosis factor (TNF) is a highly pleiotropic cytokine that plays a key role in inflammation, defense against microbial pathogens and cancer (13). Since regulation of TNFR expression is considered important in the process of inflammation, it is of interest to determine whether or not AA modulates the expression of TNF receptor (TNFR) in lymphocytes.

Esophageal carcinoma (EAC) remains the leading cause of cancer-related mortality in China. Previous studies have indicated that the generation and development of EAC are correlated with psychological stress and neuroimmunological factors (3). However, there are few studies on this topic. Here, we use AA, two types of esophageal adenocarcinoma (EAC) cell lines, SK-GT-4 and OE19, and tumor-infiltration lymphocyte (TIL) of EAC to illuminate the impact of this neurotransmitter on cell migration, necrosis, cytokine secretion and cytotoxicity of TILs. The data presented demonstrate that AA increases immigration and necrosis in tumor cells and TILs and also increases cytokine secretion and cytotoxicity of TILs. However, the extent of the increase was different in the various cells. The degree of malignancy and the ratio of regulatory T cells may be the main factors determining the role of AA.

## Materials and methods

### Reagents, cell lines and tumor tissues

The study was approved by the ethics committee of the Provincial Affiliated Hospital of Shandong University, Jinan, China. AA was purchased from Sigma-Aldrich (St. Louis, MO, USA). SDF-1 and TNF-α were purchased from R&D Systems (Minneapolis, MN, USA). The EAC cell lines, SK-GT-4 and OE19, in RPMI-1640 with 2 mM glutamine and 10% fetal bovine serum (FBS) were obtained from the American Type Culture Collection (ATCC). Fresh tumor tissues and tissue specimens were obtained from the Provincial Affiliated Hospital of Shandong University. The patients gave their consent for the cell material to be used in research. Tumor tissues were used or processed within 2 h of surgery.

### Generation of TIL

The tissues were rinsed with RPMI-1640 media containing cidomycin, penicillin and streptomycin, and were then cut into pieces. Following two washes, the tissue pieces were added to a 24-well plate, coated with anti-CD3 antibody (Beckman Coulter, Krefeld, Germany) for 2 h and cultured in RPMI-1640 media containing 10% fetal calf serum (FCS) supplemented with L-glutamine, 2-mercaptethanol and 400 U/ml interleukin (IL)-2. A part of the amplified TIL was analyzed by flow cytometry and the remaining TIL was expanded again by immobilized anti-γδT antibodies (Beckman Coulter) for generation of γδT cell-enriched TIL cells for 2 weeks. The expanded γδT cell-enriched TILs were used for cytotoxicity experiments.

### Quantitative reverse transcription-polymerase chain reaction (qRT-PCR)

qRT-PCR was carried out on a Rotor-Gene 2000 (Corbett Research, Sydney, Australia) using a SYBR-Green detection protocol (14). Total RNA was extracted from ∼5×10^6^ cells using TRIzol (Life Technologies Corporation, Carlsbad, CA, USA). Superscript II reverse transcriptase (Life Technologies Corporation) was used to generate cDNA using 1 *μ*g RNA and oligo dT primer, according to the manufacturer’s instructions. qRT-PCR was performed in triplicate and was repeated at least three times using the following conditions: reaction mixtures contained 12.5 *μ*l SYBR-Green I dye master mix (Applied Biosystems, Carlsbad, CA, USA), 2 pmol each forward and reverse primers and 5 *μ*l 100X diluted cDNA. The thermocycle conditions included initial denaturation at 50°C and 95°C (10 min each), followed by 40 cycles at 95°C (15 sec) and 60°C (1 min). Fluorescent data were acquired during each extension phase. After 40 cycles, a melting curve was generated by slowly increasing (0.1°C/sec) the temperature from 60 to 95°C. The expression levels of the genes of interest were normalised to the housekeeping control gene β-actin. The primers used were as follows: CXCR4 forward primer 5′-TCA GTG GCT GAC CTC CTC TT-3′ and reverse primer 5′-CTT GGC CTT TGA CTG TTG GT-3′; TNFR1 forward primer 5′-GGT GAC TGT CCC AAC TTT GC-3′ and reverse primer 5′-AGG CAA GTG AGG CAC CTT-3′; β-actin forward primer 5′-TCA CCC ACA CTG TGC CCA TCT ACG-3′ and reverse primer 5′-CAG CGG AAC CGC TCA TTG CCA ATG-3′.

### Immunoblotting

To evaluate the expression of CXCR4 and TNFR1, whole cell extracts following the stimulation of AA were prepared in sodium dodecyl sulfate (SDS) sample buffer containing a cocktail of protease inhibitors. Protein concentrations were determined using the Pierce bicinchoninic acid (BCA) protein assay kit (Thermo Fisher Scientific Inc., Rockford, IL, USA). Approximately 30 *μ*g total protein was used for each SDS-polyacrylamide gel electrophoresis (PAGE). The proteins were detected by incubation with the following antibodies: anti-CXCR4, anti-TNFR1 and anti-β-actin (Abcam, Cambridge, MA, USA). Antibody binding was visualized using Pierce SuperSignal West Pico, chemiluminescence (Thermo Fisher Scientific Inc.) according to the manufacturer’s instructions.

### Chemotactic transmigration assay

The target cells [5×10^5^ in 100 *μ*l RPMI/0.5% bovine serum albumin (BSA)] were added to Transwell inserts (Corning, NY, USA) with a 5-*μ*m pore size. Serially diluted recombinant SDF-1 was added to the lower chamber and target cells were allowed to migrate up to 16 h. Transwell inserts were removed and the migrated cells in the lower chamber were counted with a Coulter counter Z2 (Beckman Coulter). Migrated cells on the lower surface of the filter were detached using 100 mM ethylenediamime tetraacetic acid (EDTA) in phosphate-buffered saline (PBS) and counted with the Coulter Counter Z2.

### Cell death assay

SK-GT-4 and OE19 cells were treated with TNF-α (5 ng/ml). TNF-α was used as a necrosis-inducing agent. Before adding TNF-α, the SK-GT-4 and OE19 cells were treated with AA at 5, 10, 20 and 40 *μ*M, respectively, for 12 h. The CellTiter-Glo assay was performed according to the manufacturer’s instructions (G7570, Promega, Madison, WI, USA). Luminescence was measured using a Synergy HT machine (Biotek Instruments Inc., Winooski, VT, USA).

### Cytokine concentration detection

Cytokine concentrations in cell-free supernatants were determined following cell stimulation with plate-bound anti-CD3 and anti-CD28 monoclonal antibodies (MAbs; 10 *μ*g/ml; BD Pharmingen, San Diego, CA, USA). Supernatants were collected after 48 h of culture and analyzed for cytokine content using specific enzyme-linked immunosorbent assay (ELISA) kits (R&D Systems), following the manufacturer’s instructions.

### Immunofluorescent analysis by flow cytometry

To analyze the expression of T cell receptor (TCR)-γδ and TCRαβ on TILs, cells were stained with phycoecrythrin (PE)-anti-TCRαβ or fluorescein isothiocyanate (FITC)-anti-TCRγδ and the corresponding isotype controls (Beckman Coulter). To analyze the expression of forkhead box p3 (Foxp3) on TILs, cells were stained with PE-anti-Foxp3 or FITC-anti-CD4 and the corresponding isotype controls (Beckman Coulter). The cells were analyzed using a flow cytometer (BD Biosciences, San Jose, California, USA). Immunofluorescence was measured by Accuri™ C6 flow cytometer and analyzed by CFlow software.

### Cytotoxicity assay

SK-GT-4 and OE19 as target cells were added respectively to the 96-well plates at a density of 3×10^4^ per well. TILs were incubated with AA at 10, 20 and 30 *μ*M or alone for 12 h at 37°C and incubated with an anti-γδTCR antibody, isotype IgG1 antibody or alone for 1 h at 4°C. They were then added to the plate at effector/target ratios of 2.5:1, 5:1 and 10:1, respectively, and each condition was plated in triplicate. There were four control groups: maximal cpm release group, volume corrected group, background group and spontaneous cpm release group. The cytotoxicity was detected according to the manufacturer’s instructions for the 96 non-radioactive cytotoxicity assay kit (Promega) (15).

## Results

### Effectiveness of AA on migration and necrosis of SK-GT-4 and OE19 cells

**Figure 1:**
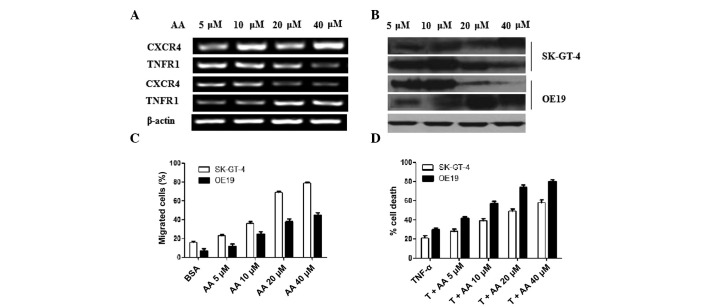
Effectiveness of AA on migration and necrosis of SK-GT-4 and OE19 cells. (A) mRNA expression of CXCR4, TNFR1 and β-actin were detected through agarose gel electrophoresis (AGE) following qRT-PCR. The concentrations of AA used to stimulate the tumor cells were 5, 10, 20 and 40 *μ*M. (B) Protein expression of CXCR4, TNFR1 and β-actin were detected through western blotting. The same protein concentration for each sample was loaded. (C) Chemotactic responses (CTR) of OE19 and SK-GT-4 cells were detected in the presence of AA. After adding chemotactic factor SDF-1, the migration of SK-GT-4 cells increased more clearly than OE19 cells with increased AA. Data were presented as mean ± SD of three similar experiments run in triplicate. (D) Cell death assay was carried out to evaluate the effectiveness of AA on necrosis. OE19 and SK-GT-4 cells with or without AA induction were treated with TNF-α (T) (5 ng/ml) for 24 h. The experimental results were divided by the control results to calculate the percentage of cell death. Cell viability was determined using the CellTiter-Glo assay. Data are presented as mean ± SD of duplicates. AA, arachidonic acid; CXCR4, C-X-C chemokine receptor type 4; TNFR4, tumor necrosis factor receptor 4; qRT-PCR, quantitative reverse transcription-polymerase chain reaction; SDF-1, stromal cell-derived factor 1; SD, standard deviation; TNF, tumor necrosis factor; BSA, bovine serum albumin.

One type of migration is a non-genetic regulation of metastasis formation. SDF-1 and its receptor CXCR4 are notable targets for this process. Hence, here we investigated CXCR4 expression and chemotactic transmigration assay to evaluate the effectiveness of AA on migration. AA as a neurotransmitter may induce inflammation. Inflammation induces necrosis through multiple pathways (16) and TNF and its receptor TNFR is one of the associated pathways. We investigated TNFR1 expression and the percentage of cell death in the presence of TNF-α to evaluate the effect of AA on necrosis. The results are summarized in Fig. 1. qRT-PCR was used to compare the difference in CXCR4 and TNFR1 mRNA expression in OE19 and SK-GT-4 cells with an increase of AA. The mRNA levels of CXCR4 in SK-GT-4 cells increased dose-dependently, while TNFR1 decreased dose-dependently. This was reversed in OE19 cells (Fig. 1A). Western blotting was performed to evaluate the protein expression of CXCR4 and TNFR1. The change in CXCR4 and TNFR1 protein level was coincident with the change in mRNA level. β-actin was used as a quantitative reference (Fig. 1B). The migration of SK-GT-4 and OE19 cells increased gradually with increased AA after adding SDF-1. Furthermore, the increase of SK-GT-4 cells was more apparent than OE19 cells (Fig. 1C). SK-GT-4 cells were prone to migration on addition of AA. Adding AA inhibited CXCR5 mRNA expression; however, it did not inhibit the migration of OE19 cells. Metastasis is a sign of poor prognosis. These results indicate that AA may aggravate EAC, whose characteristics are similar to SK-GT-4 cells.

After adding AA, the necrosis induced by TNF-α was accelerated (Fig. 1D). The cell death percentage of OE19 and SK-GT-4 cells increased dose-dependently with AA in the existence of TNF-α. Furthermore, the cell death percentage of OE19 cells was higher than SK-GT-4 cells. This indicates that AA accelerates necrosis of OE19 cells more easily than SK-GT-4 cells. We identified that the expression of TNFR1 in OE19 cells without AA stimulation was the lowest and as the levels of AA increased, the expression of TNFR1 increased (Fig. 1A). This indicates that the TNF pathway was inhibited in OE19 cells and AA blocks this inhibition, activating the TNF pathway to enhance necrosis. Necrosis is necessary for tumor therapy. *In vivo*, necrotic cell death is extremely pro-inflammatory. When cancer cells die from necrosis in response to chemotherapy, they activate the innate immune response and possibly, if there are cancer-specific antigens, a response against the remaining cancer cells, providing a potential way for the immune system to actively deal with the remaining cancer cells (16). Therefore, AA may attenuate EAC, whose characteristics are similar to OE19 cells.

### Effectiveness of AA on migration and necrosis of TILs

**Figure 2:**
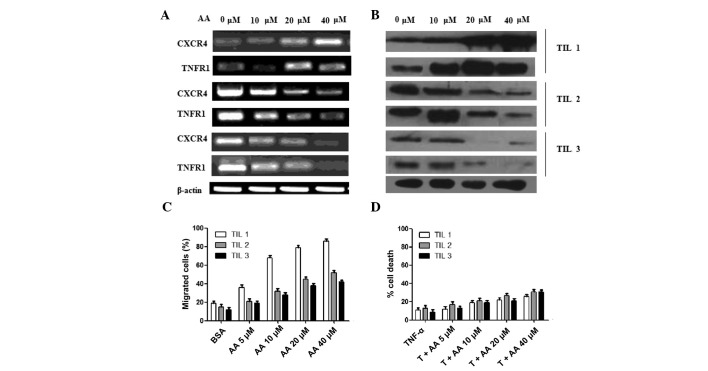
Effectiveness of AA on migration and necrosis of TILs derived from three EAC patients named TIL1, TIL2 and TIL3. (A) mRNA expression of CXCR4, TNFR1 and β-actin was detected through agarose gel electrophoresis (AGE) following qRT-PCR. The concentrations of AA used to stimulate the tumor cells were 5, 10, 20 and 40 *μ*M. (B) Protein expression of CXCR4, TNFR1 and β-actin were detected through western blotting. The same protein concentration for each sample was loaded. (C) Chemotactic responses (CTR) of the three TILs were detected in the presence of AA. After adding chemotactic factor SDF-1, the migration of OE19 cells increased more clearly than SK-GT-4 cells with increased AA. Data were presented as mean ± SD of three similar experiments run in triplicate. (D) A cell death assay was performed to evaluate the effectiveness of AA on necrosis. The three TILs with or without AA induction were treated with TNF-α (T; 5 ng/ml) for 24 h. The experimental results were divided by the control results to calculate the percentage of cell death. Cell viability was determined using the CellTiter-Glo assay. The percentage of cell death was calculated by determining the percentage of viable cells: percentage of viable cells = (percentage of TNF-α and AA reagent-treated cells) / (percentage of control-treated cells) ×100. Percentage of cell death = 100 − percentage of viable cells. Data are presented as mean ± SD of duplicates. AA, arachidonic acid; TILs, tumor-infiltrating lymphocytes; CXCR4, C-X-C chemokine receptor type 4; TNFR4, tumor necrosis factor receptor 4; qRT-PCR, quantitative reverse transcription-polymerase chain reaction; SDF-1, stromal cell-derived factor 1; SD, standard deviation; TNF, tumor necrosis factor; BSA, bovine serum albumin.

To evaluate the effectiveness of AA on lymphocytes, TILs were derived from three esophageal carcinoma patients named TIL1, TIL2 and TIL3. The mRNA and protein expression of CXCR4 and TNFR1 are shown in Fig. 2A and B. The change in trend of CXCR4 and TNFR1 in TILs was similar. In TIL1, CXCR4 and TNFR1 increased dose-dependently with AA. In TIL2 and TIL3, they decreased dose-dependently with AA. The migration of TILs was also detected in the existence of SDF-1. The migration of the three TILs increased dose-dependently with increasing levels of AA. Furthermore, the increase of TIL1 was more apparent than TIL2 and TIL3 (Fig. 2C). The enhancement of necrosis of TILs with AA was also detected in the presence of TNF-α (Fig. 2D). AA weakly increased the percentage of cell death of the three TILs. This result was different to the tumor cells (Fig. 1D).

### Phenotype and cytokine secretion of TILs derived from EAC patients

**Figure 3:**
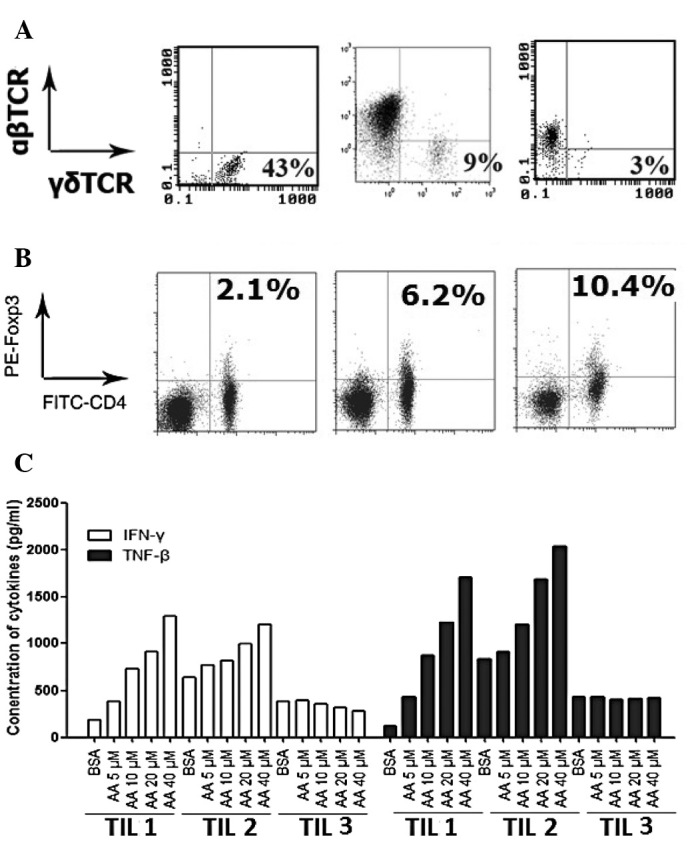
Phenotype of the TILs and the secretion of IFN-γ and TNF-β. (A) The percentages of αβT cells and γδT cells in the three TILs were analyzed by flow cytometry. FITC-anti-TCRγδ and PE-anti-TCRαβ were used. (B) The percentages of CD4^+^Foxp3^+^ regulatory T cells in the three TILs were analyzed by flow cytometry. FITC-anti-CD4 and PE-anti-Foxp3 were used. (C) The concentrations of IFN-γ and TNF-β in the culture supernatant of the three TILs were detected using cytokine quantitative ELISA kits. TILs, tumor-infiltrating lymphocytes; IFN, interferon; TNF, tumor necrosis factor; FITC, fluorescein isothiocyanate; TCR, T cell receptor; PE, phycoecrythrin; Foxp3, forkhead box p3; ELISA, enzyme-linked immunosorbent assay.

To evaluate the effectiveness of AA on lymphocytes in tumor patients, three EAC patients were randomly selected and their lymphocytes in tumor tissues were cultured and activated by anti-CD3 monoclonal antibody. The percentages of αβT cells and γδT cells in the three TILs were analyzed by flow cytometry (Fig. 3A). The numbers on the figure are the percentages of γδT cells. TIL1 contained 43% γδT cells and 57% αβT cells, TIL2 contained 3% γδT cells and 97% αβT cells and TIL3 contained 9% γδT cells and 91% αβT cells. The percentages of regulatory T cells in the three TILs were also analyzed (Fig. 3B). The numbers on the figure are the percentages of CD4^+^Foxp3^+^ regulatory T cells. The three TILs were stained by anti-Foxp3 and anti-CD4. The percentages of CD4^+^Foxp3^+^ regulatory T cells in TIL1, TIL2 and TIL3 were 2.1, 6.2 and 10.4%, respectively.

To investigate the effectiveness of AA on cytokine secretion in the three TILs, the secretion of IFN-γ and TNF-β were detected. Following activation by anti-CD3 for 2 weeks, the culture supernatants of the three TILs were collected to detect the concentration of IFN-γ and TNF-β. The results are summarized in Fig. 3C. AA dose-dependently increased IFN-γ and TNF-β secretion in TIL1 and TIL2. However, AA did not increase IFN-γ and TNF-β secretion in TIL3.

### Cytotoxicity of three γδT cell-enriched TILs against OE19 and SK-GT-4 cells

**Figure 4:**
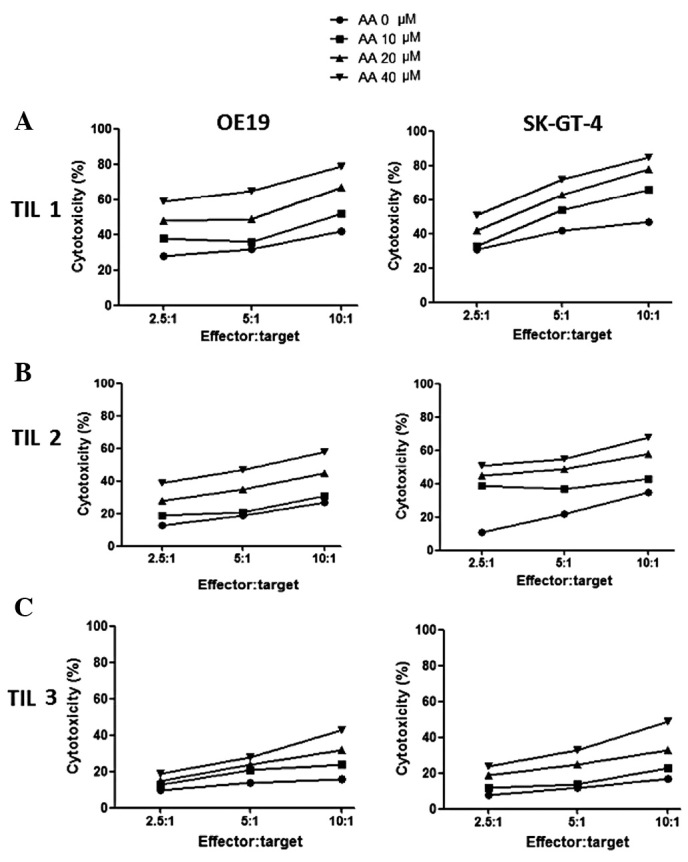
Cytotoxicity assays of the three TILs compared to SK-GT-4 or OE19 cells. OE19 cells are shown in the left column and SK-GT-4 cells are shown in right column. TILs were treated with AA at the indicated concentration for 24 h. The cytotoxicity was determined using the cytotoxicity detection kit. The ratios of effector-to-target cells were 2.5:1, 5:1 and 10:1. Data are representative of three independent experiments. TILs, tumor-infiltrating lymphocytes; AA, arachidonic acid.

To further evaluate the effectiveness of AA on antitumor activities of lymphocytes, the cytotoxicities of the three γδT cell-enriched TILs stimulated by AA at various concentrations were detected. The results are summarized in Fig. 4. The target cells in the left and right columns are the cell lines OE19 and SK-GT-4, respectively. The effector cells in the first, second and last rows are TIL1, TIL2 and TIL3, respectively. In general, the cytotoxicity of three γδT cell-enriched TILs compared to OE19 and SK-GT-4 cells increased dose-dependently with the increasing levels of AA. The cytotoxicity of γδT cell-enriched TIL1 compared to OE19 and SK-GT-4 cells was the highest, while the cytotoxicity of γδT cell-enriched TIL3 compared to OE19 and SK-GT-4 cells was the lowest. Furthermore, there was no difference in the ability to kill OE19 and SK-GT-4 cells for each TIL. Our results indicate that γδT cell-enriched TIL1 is more effective at killing EAC cells while γδT cell-enriched TIL3 is less effective at killing EAC cells. This may be due to the different percentages of regulator T cells in TILs, which have different responses to AA. This indicates that AA may magnify cytotoxicity effectiveness of TILs.

## Discussion

In this study, the effectiveness of AA on two types of EAC cell lines, SK-GT-4 and OE19, as well as TILs was observed. AA acted as an accelerator of cell migration, necrosis, cytokine secretion and cytotoxicity. AA dose-dependently increased the migration of lymphocytes and tumor cells. However, AA only increased tumor cell necrosis. The cytotoxicity and cytokine secretion of TIL3 was the lowest. This may be due to the fact that the percentage of regulatory T cells in TIL3 was the highest.

The function of AA depends on the degree of tumor malignancy. From the results of cell migration, necrosis and cytokine secretion, it is indicated that AA aggravates OE19 cells, and attenuates SK-GT-4 cells. SK-GT-4 cells were established from a primary tumor in 1989 from a 89-year-old Caucasian male who presented with dysphagia secondary to a well-differentiated adenocarcinoma arising in the Barrett epithelium of the distal esophagus. The tumor invaded into, but not through the muscle layer and involved 3 of 14 lymph nodes. SK-GT-4 cells were identified to be tumorigenic in athymic nu/nu mice. The cell line OE19, also known as JROECL19, was established in 1993 from an adenocarcinoma of the gastric cardia/esophageal gastric junction of a 72-year-old male patient. The tumor was identified as pathological stage III [Union for International Cancer Control (UICC)] and demonstrated moderate differentiation. OE19 cells presented milder malignancy than SK-GT-4 cells. This indicates that AA may promote tumor metastasis in severe malignant EAC more easily than in mild malignant EAC and AA may promote tumor necrosis in mild malignant EAC more easily than in severe malignant EAC. However, this should be validated in more tumor cell lines.

The function of AA depends on the percentage of regulatory T cells in TILs. The number of Foxp3^+^ Treg within human tumors is correlated with a poorer prognosis. Patients with ovarian or gastric cancer and lower numbers of Treg TILs have improved disease-specific survival (17). In this study, three types of TILs were derived from EAC patients. TIL1 contained the highest number of γδT cells and the lowest number of regulatory T cells, while TIL3 contained the lowest number of γδT cells and the highest number of regulatory T cells. AA increased the migration of TIL1 and the cytotoxicity of γδT cell-enriched TIL1. However, cytokine secretion of TIL3 and the cytotoxicity of γδT cell-enriched TIL3 was lower in the presence of AA. In this study, regulatory T cell analysis was performed and the secretion of IFN-γ and TNF-β of TILs was detected prior to γδT cell expansion in TILs. Before γδT cells were expanded, TIL3 secreted few IFN-γ and TNF-β, and after γδT cells were expanded, the cytotoxicity of γδT cell-enriched TIL3 remained the lowest. This indicates that γδT cells contained fewer CD4^+^Foxp3^+^ regulatory T cells than αβT cells. From this study, we conclude that regulatory T cells are insensitive to responses of AA.

Necrosis is a useful method for tumor cell death and is regulated by AA. AA aggravated OE19, while AA attenuated SK-GT-4 cells. During carcinogenesis, there is a drive in the cancer cells to acquire mutations that render them resistant to apoptosis. Cancer cells die from necrosis in response to chemotherapy. This may activate the innate immune response and possibly, if there are cancer-specific antigens, activate a response against the remaining cancer cells, providing a potential method for the immune system to actively remove the remaining cancer cells. In this study, TILs were insensitive to the responses of AA in the presence of TNF-α. It is safe to use necrosis-inducing agents *in vivo* for tumor therapy, as they do not damage immune cells.

In conclusion, this study investigated the regulation of AA in tumor cells and lymphocytes. Facilitative functions of AA on cell migration, necrosis, cytokine secretion and cytotoxicity were shown. The degree of malignancy of tumors and the ratio of regulatory T cells may be the main factors determining the function of AA.
